# Predicting hypoglycemia risk after gastrointestinal surgery in type 2 diabetes mellitus: a retrospective cohort study

**DOI:** 10.3389/fendo.2025.1590780

**Published:** 2025-07-07

**Authors:** Huilan Yao, Shijin Yuan, Hongying Pan, Sisi Hong, Chen Huang, Linfang Zhao, Hongdi Yuan, Lei Mei, Yinghong Zheng, Xiaolong Liu, Weina Lu

**Affiliations:** ^1^ Nursing Department, Sir Run Run Shaw Hospital, Zhejiang University School of Medicine, Hangzhou, Zhejiang, China; ^2^ Department of Medical Oncology, Sir Run Run Shaw Hospital, Zhejiang University School of Medicine, Hangzhou, Zhejiang, China; ^3^ Department of General Surgery, Sir Run Run Shaw Hospital, Zhejiang University School of Medicine, Hangzhou, Zhejiang, China; ^4^ Department of Endocrinology, Sir Run Run Shaw Hospital, Zhejiang University School of Medicine, Hangzhou, Zhejiang, China

**Keywords:** gastrointestinal tumor, diabetes, surgery, hypoglycemia, risk factors, prediction mode

## Abstract

**Objective:**

To identify factors influencing hypoglycemia in patients with type 2 diabetes mellitus (T2DM) following gastrointestinal tumor surgery and construct a predictive model for assessing hypoglycemia risk.

**Methods:**

We retrospectively collected data on 1280 patients with T2DM who underwent gastrointestinal tumor surgery and divided them into two groups—one for model building (n = 982) and another for validation (n = 298). We used multivariate logistic regression to develop a predictive model for hypoglycemia following gastrointestinal tumor surgery. The model’s predictive performance was evaluated using the area under the receiver operating characteristic (ROC) curve, and its generalization ability was evaluated using the bootstrap test and the five-fold cross-validation test.

**Results:**

We identified hypoglycemia following gastrointestinal tumor surgery in 124 of 982 (12.6%) T2DM patients in the developmental cohort. Finally, five predictors, including duration of diabetes, operation duration, preoperative fasting time, preoperative hypoglycemic regimen (subcutaneous insulin injection), and glucose fluctuation on the day of surgery, were integrated into the predictive model. The performance of the hypoglycemia risk prediction model for patients with T2DM undergoing gastrointestinal tumor surgery was comprehensively evaluated. The model demonstrated an area under the ROC curve (AUC) of 0.837 (95% CI: 0.792–0.882), indicating a strong discriminative ability. Internal validation via five-fold cross-validation with bootstrap resampling revealed close approximation of the calibration curve to the ideal line, refining high consistency between predicted probabilities and actual hypoglycemia occurrence. Decision curve analysis (DCA) further supported its clinical utility, indicating value in clinical decision making for hypoglycemia risk stratification and preventive intervention selection.

**Conclusion:**

The developed model exhibits high discriminative ability and good calibration. Following visualization (e.g., nomogram), it provides a clinical tool for healthcare providers to stratify hypoglycemia risk in T2DM patients undergoing gastrointestinal tumor surgery, enabling personalized perioperative glucose management and informed decision making to improve patient outcomes.

## 1 Introduction

Gastrointestinal tumors are the most common malignancies in China, accounting for nearly half of the global cases, and their incidence and mortality rates have been rising in recent years (1, 2). Diabetes mellitus (DM) is a major global health issue with high prevalence, disability, mortality, and disease burden. By 2025, approximately 784 million adults (20–79 years) are estimated to have DM, with type 2 DM (T2DM) constituting over 90% of cases (3–5). China has the highest number of DM patients, with an estimated 140 million adults affected and a prevalence rate of around 12.4% (3). Epidemiological studies have shown that T2DM is associated with an increased risk of gastric, colorectal, and rectal cancer (6). As the number of T2DM patients requiring gastrointestinal tumor surgery increases, they face a 5–6 times higher risk of postoperative complications and mortality compared to non-diabetic patients (7). While postoperative blood glucose control can reduce complications, it may lead to hypoglycemia (8). Hypoglycemia is a common complication in T2DM patients, with incidence rates ranging from 3.3% to 24.78% (9, 10). Perioperative hypoglycemia is associated with a range of adverse outcomes, including cognitive dysfunction, brain damage, cardiovascular events, increased morbidity, and mortality (11).

The occurrence of hypoglycemia is the result of many factors. There are many factors that influence postoperative hypoglycemia in T2DM patients. According to the current advances, these influencing factors can be generally divided into several avenues: individual factors of patients, such as old age, female, low body mass index, long course of diabetes, mental state (anxiety, depression, fear), and unhealthy lifestyle of smoking and drinking; surgical factors, such as open surgery, prolonged operation time, prolonged fasting, prolonged parenteral nutrition, and blood glucose variability; disease factors, such as cardiovascular disease, renal insufficiency, and abnormal liver function; and drug factors, such as improper administration of hypoglycemic drugs before surgery, excessive dose or improper administration time of insulin after surgery, etc. (12–15).

In addition, studies demonstrate that digestive tract reconstruction and modified surgeries after surgery for gastrointestinal tumor resection can alleviate T2DM symptoms in gastric cancer patients and improve glucose metabolism (16). The mechanism may involve altered secretion of gastrointestinal hormones and reduced functional digestive tract volume (17). Such procedures as Roux-en-Y gastric bypass (RYGB) can significantly impact glucose metabolism and insulin sensitivity, thereby influencing blood glucose levels. These surgeries can alter the secretion of gastrointestinal hormones, leading to rapid carbohydrate absorption and increased secretion of hormones like glucagon-like peptide-1 (GLP-1) and peptide YY (PYY), which in turn affect insulin secretion and gastric emptying. This can result in postprandial reactive hypoglycemia. Moreover, the surgeries can also impair gastric function and appetite regulation, potentially increasing the risk of fasting hypoglycemia (18). Additionally, the stress associated with surgery can induce oxidative stress, which stimulates the secretion of adropin, a peptide involved in regulating glucose metabolism. Adropin enhances insulin sensitivity by boosting hepatic AKT/PKB signaling and increasing GLUT4 expression in skeletal muscle, promoting glucose uptake and utilization. It also interacts with other hormones to maintain glucose homeostasis. However, the complex interplay of these factors can sometimes lead to hypoglycemia, especially in the postoperative period (19). Therefore, understanding these mechanisms is crucial for managing and preventing hypoglycemia in patients undergoing gastrointestinal surgery. However, few studies have focused on the risk of postoperative hypoglycemia in patients with T2DM undergoing this type of surgery.

Presently, numerous researchers worldwide have developed risk prediction models for hypoglycemia in patients with T2DM (20–23), including a predictive model for the risk of postoperative hypoglycemia in patients with T2DM undergoing elective surgery (9, 24). However, these studies lack clarification on the predictive time window, which diminishes their clinical applicability, and they did not explore the impact of different types of surgery on the probability of hypoglycemia. Our previous study showed that 8.37% of patients with T2DM developed hypoglycemia during gastrointestinal tumor surgery. Ideally, a model that could precisely predict postoperative hypoglycemia in T2DM patients undergoing a specific surgery type would exhibit superior specificity and efficiency. Therefore, we conducted this study to develop a risk prediction model for hypoglycemia in patients with T2DM following gastrointestinal tumor surgery.

In summary, the growing prevalence of gastrointestinal tumors and T2DM in China highlights the need for a deeper understanding of the pathophysiological mechanisms underlying postoperative hypoglycemia in patients undergoing gastrointestinal surgery. This understanding is crucial for developing effective strategies to manage and prevent hypoglycemia in this vulnerable patient population.

## 2 Materials and methods

### 2.1 Study design

We conducted a retrospective cohort observational study of patients with T2DM admitted for gastrointestinal tumor surgery between January 1, 2018, and February 28, 2023, using data from the hospital’s electronic information system. According to the inclusion and exclusion criteria, 1280 patients with T2DM were enrolled. Inclusion criteria were as follows: adult patients (≥18 years) diagnosed with T2DM, according to the International Classification of Diseases, Tenth Revision (ICD-10: E11), who underwent gastrointestinal tumor surgery. Exclusion criteria included (1) patients undergoing emergency surgery, multiple surgeries, or canceled surgeries; (2) those with severe heart, lung, liver, or kidney diseases; (3) those with other malignancies or simultaneous radical surgeries for non-gastrointestinal tumors; and (4) those with incomplete or unanalyzable clinical, laboratory, or surgical data.

### 2.2 Data collection

To ensure the research’s scientific validity, authority, and feasibility, we organized experts with extensive clinical experience to discuss the risk predictors of hypoglycemia found in the literature, determining their significance and relevance. The inclusion criteria were professionals in diabetes and gastrointestinal tumor surgeries (doctors, nursing managers, nursing educators, and diabetes specialist nurses), over 10 years’ experience in endocrinology or general surgery, significant academic influence in diabetes or nursing, and voluntary participation with informed consent. Ultimately, 12 experts were selected: 16.6% doctors (1 general surgeon and 1 endocrinologist), 16.6% nursing managers (1 deputy nursing director and 1 general surgery head nurse), 50% specialized nursing unit nurses (3 endocrinology nurses, 1 ICU nurse, and 2 general surgery nurses), and 16.6% senior diabetes clinical specialist nurses (2 certified diabetes specialist nurses). Possible new impact factors have even been proposed in the discussion. Finally, data were collected based on the factors influencing hypoglycemia experts discussed.

Initial findings from our research indicated that about 30% of hypoglycemia occurred on the 2nd to 8th day after surgery. Hence, the researchers determined that the model should be applied on the 1st day after surgery to identify high-risk patients and propose timely intervention measures. Based on clinical expertise and expert discussion results, the influencing factors of hypoglycemia were summarized into three time points: before, on the day of surgery, and the day after surgery.

The researchers designed a data collection table to collect information on factors influencing hypoglycemia based on expert discussion results, including patient age, sex (male or female), hypertension (yes or no), renal impairment (yes or no), personal history(smoking or drinking), preoperative body mass index, preoperative fasting(yes or no), preoperative nutritional support, duration of diabetes, preoperative metabolic indicators (total cholesterol, triglycerides), preoperative HbA1C, preoperative hypoglycemic medication administration methods. Type of gastrointestinal reconstruction (yes or no), time of gastrointestinal tumour surgery, Intraoperative use of hypoglycemic drugs (yes or no), glycemic variability on the day of surgery, postoperative hypoglycemic medication administration methods, glycemic variability over multiple days(glycemic variability from admission to postoperative day 1).

### 2.3 Definitions of clinical endpoints

The clinical endpoint of our study was the occurrence of hypoglycemia following gastrointestinal tumor surgery, defined as a blood glucose level <70 mg/dL (3.9 mmol/L) (25). Our study measured blood glucose using point-of-care capillary blood glucose testing with standard hospital-grade glucometers (OneTouch VerioVue). All glucometers undergo regular calibration and quality control checks by our hospital’s standard operating procedures, and all measurements are performed by trained nursing staffs. Hypoglycemia was determined based on documentation from the Nursing Electronic Case and Hospital Information System.

### 2.4 Sample size calculation

This is a retrospective cohort study aimed at establishing a risk prediction model for hypoglycemia in patients with T2DM after gastrointestinal tumor surgery. Therefore, calculating the target sample size for modeling primarily refers to the method used for estimating the sample size in the risk prediction model. Sun Yaqing considered the number of events in the outcome variable to be 5–10 times the number of independent variables included in the model (26). In this risk prediction model analysis, we intend to include 16 variables. In the training cohort of this study model, 109 patients experienced hypoglycemia events, meeting the study’s requirements. In 2022, a survey found that the incidence of hypoglycemia in T2DM patients after gastrointestinal surgery was 8.37% in our hospital. So, the total adequate sample size required for the model construction part of this study was 956 cases. There were 982 patients who were suitable for modeling analysis, and 298 were validated.

### 2.5 Statistical analysis

Data processing and analysis were performed using SPSS software (version 22.0) and R software (version 3.6.0). Statistical tests were two-sided, with a *P* value of <0.05 indicating statistical significance. We used the method of mean imputation to deal with missing data. Missing data included HbA1c (1.42%), total cholesterol (0.70%), body mass index (0.67%), and triglycerides (0.70%). We describe continuous data in terms of median (quartile) and categorical data in terms of frequency (%). Pearson Chi-square tests or Fisher precision tests evaluated categorical variables. The Mann-Whitney U test was used to analyze continuous variables. Risk factors were initially identified through single-factor logistic regression (*p* < 0.05) and then entered into multivariate logistic regression analysis. Forward stepwise regression was used to select the variables that eventually entered the model. The receiver operating characteristic (ROC) analysis and calibration plots were used to evaluate the model’s discrimination (ability to distinguish between patients with and without hypoglycemia risk) and calibration (consistency between predicted and observed hypoglycemia risk probabilities), respectively. The clinical usefulness of the nomogram was assessed using decision curve analysis.

### 2.6 Development and validation of the hypoglycemia model

The data included the period from January 2018 to August 2022 as the development cohort (n = 982), as data from other institutions were not available. Therefore, data from September 2022 to February 2023 were selected to complete external verification (n = 298).

Univariate analyses of all descriptive variables were performed with the occurrence of hypoglycemia (yes/no) as the dependent variable at a significance level of *p* < 0.10 to screen candidates for multivariate analysis. Subsequently, independent variables were identified through multivariate logistic regression analysis (using a backward stepwise approach), and a predictive model for hypoglycemia following gastrointestinal tumor surgery in T2DM patients was constructed. Based on the multivariate analysis results, a nomogram was created to predict the probability of hypoglycemia after gastrointestinal tumor surgery in patients with T2DM. We utilized the bootstrap resampling method (with 1000 bootstrap resamples) and a five-fold cross-validation test for internal validation of the cohort. External validation of the nomogram was performed using an external validation cohort. Data collection and verification for this external cohort followed the same methodology as that employed for the development cohort.

## 3 Results

### 3.1 Patient characteristics

There were 1280 patients enrolled in this study, including a development cohort of 982 patients and a validation cohort of 298 patients. The partial essential characteristics of the subjects in the development cohort were as follows: 647 men (65.9%) and 335 women (34.10%), with 124 cases (12.6%) experiencing hypoglycemia. In the validation cohort, there were 203 men (68.10%) and 95 women (31.9%), with 15 cases of hypoglycemia accounting for 11.1%. The demographic characteristics of patients in the development and validation cohorts were consistent and showed no statistically significant differences. **Table 1** delineates the patient characteristics.

**Table 1:** General clinical characteristics and related variables of patients.

Variable	Total (n = 1280)	Developing cohort	Validation cohort	*P* value
		(n = 982)	(n = 298)	
Sex:				0.692
Male	850 (66.4%)	647 (65.9%)	203 (68.1%)	
Female	430 (33.6%)	335 (34.1%)	95 (31.9%)	
Age group (y):				0.108
18–34	9 (0.70%)	7 (0.7%)	2 (0.7%)	
35–59	221 (17.3%)	166 (16.9%)	55 (18.5%)	
≥60	1050 (82.0%)	809 (82.4%)	241 (80.8%)	
Drink:				0.389
No	969 (75.7%)	739 (75.3%)	230 (77.2%)	
Yes	311 (24.3%)	243 (24.7%)	68 (22.8%)	
Smoking:				0.462
No	964 (75.3%)	736 (74.9%)	228 (76.5%)	
Yes	316 (24.7%)	246 (25.1%)	70 (23.5%)	
Hypertension:				0.548
No	843 (65.9%)	643 (65.5%)	200 (67.1%)	
Yes	437 (34.1%)	339 (34.5%)	98 (32.9%)	
Duration of diabetes	1.00 [1.00; 5.00]	1.00 [1.00; 5.00]	1.00 [1.00; 5.00]	0.379
Kidney disease:				1
No	1269 (99.1%)	974 (99.2%)	295 (99.0%)	
Yes	11 (0.9%)	8 (0.8%)	3 (1.0%)	
Digestive tract bypass reconstruction				0.563
No	953 (74.5%)	727 (74.1%)	226 (75.8%)	
Yes	327 (25.5%)	255 (25.9%)	72 (24.2%)	
Hypoglycemic administration methods:				0.894
Others	1160 (90.6%)	888 (90.4%)	272 (91.3%)	
Subcutaneous	111 (8.7%)	87 (8.87%)	24 (8.03%)	
Taken orally	9 (0.70%)	7 (0.71%)	2 (0.67%)	
Intraoperative hypoglycemic administration methods:				0.681
No	1273 (99.46%)	976 (99.4%)	297 (99.66%)	
Bolus insulin	7 (0.54%)	6 (0.60%)	1 (0.34%)	
Hypoglycemic agents were used 1 day after surgery:				1
Intravenous infusion	1216 (95.0%)	933 (95.0%)	283 (94.97%)	
Subcutaneous injection	64 (5.0%)	49 (4.99%)	15 (5.03%)	
Preoperative nutritional support mode:				0.214
No	159 (12.4%)	126 (12.83%)	33 (11.0%)	
Parenteral	343 (26.8%)	254 (25.86%)	89 (30.0%)	
Enteral	88 (6.9%)	66 (6.72%)	22 (7.4%)	
Enteral+Parenteral	690 (53.9%)	536 (54.6%)	154 (51.6%)	
Preoperative fasting time:				0.772
>24 h	1089 (85.1%)	834 (84.9%)	255 (85.6%)	
≤24 h	191 (14.9%)	148 (15.1%)	43 (14.4%)	
The standard deviation of blood glucose levels over multiple days	2.00 [1.38; 2.90]	2.04 [1.40; 2.86]	1.92 [1.28; 2.99]	0.576
Maximum range of blood glucose fluctuations over multiple days	6.50 [4.00; 10.0]	6.60 [4.00; 9.88]	6.00 [3.90; 10.4]	0.453
Blood glucose fluctuation on the day of surgery	−1.00 [−1.00; 0.5]	−1.00 [−1.00; 0.58]	−1.00 [−1.00; 0.44]	0.926
Operation duration (h)	210 [170; 270]	210 [170; 274]	210 [172; 260]	0.579
TC (mmol/L)	4.29 [3.54; 5.08]	4.28 [3.55; 5.05]	4.37 [3.54; 5.14]	0.568
TG (mmol/L)	1.23 [0.92; 1.68]	1.23 [0.92; 1.70]	1.23 [0.91; 1.66]	0.518
HbA1C (%)	6.10 [5.50; 6.80]	6.10 [5.50; 6.80]	6.10 [5.60; 6.77]	0.392
BMI (kg/m^2^)	22.5 [20.5; 24.9]	22.5 [20.4; 24.8]	22.8 [20.8; 24.9]	0.242
The maximum amplitude of blood glucose fluctuation on the day of surgery	0.00 [0.00; 1.00]	0.00 [0.00; 1.00]	0.00 [0.00; 0.90]	0.965

Values are presented as n (%) and median (IQR); TC, total cholesterol; HbA1c, glycated hemoglobin; TG, triglyceride; BMI, body mass index.

### 3.2 Predictors through univariate and multivariate logistic regression analysis

We compared the intergroup differences between the non-hypoglycemic and hypoglycemic groups in the developmental cohort. As shown in **Table 2**, duration of diabetes, operation duration, total cholesterol, glycated hemoglobin, preoperative hypoglycemic administration methods and whether intravenous insulin during surgery were used, preoperative fasting time, use of hypoglycemic agents 1 day after surgery, and blood glucose variability (including the standard deviation of multi-day blood glucose levels, maximum range of blood glucose fluctuations over multiple days, blood glucose fluctuation on the day of surgery, maximum amplitude of blood glucose fluctuation on the day of surgery) were entered into multivariate analysis as candidate variables (*p* < 0.005). It is worth noting that age (*p* = 0.089) showed a trend toward significance, suggesting a potential association with the risk of hypoglycemia, although this association was not statistically significant. The method of gastrointestinal reconstruction (*p* = 0.154) was not significantly related to hypoglycemia.

**Table 2:** Univariate analysis of influencing factors on the risk of hypoglycemia after gastrointestinal tumor surgery in patients with T2DM.

Variable	Developing cohort (n = 982)	No hypoglycemia (n = 858)	Hypoglycemia occurred (n = 124)	*P* value
Sex:				0.484
Male	647 (65.90%)	559 (65.20%)	88 (69.30%)	
Female	335 (34.10%)	299 (34.80%)	36 (30.70%)	
Age group:	72.0 [62.0; 78.0]	72.0 [62.0; 78.0]	74.0 [65.0; 79.0]	0.089
Drink:				0.214
No	739 (75.3%)	639 (74.50%)	100 (84.60%)	
Yes	243 (24.7%)	219 (25.50%)	24 (19.40%)	
Smoke:				0.935
No	736 (74.9%)	640 (74.60%)	96 (77.40%)	
Yes	246 (25.1%)	218 (25.40%)	28 (22.60%)	
Hypertension:				0.343
No	639 (65.10%)	565 (65.85%)	74 (59.70%)	
Yes	343 (34.90%)	293 (34.15%)	50 (40.30%)	
Duration of diabetes	1.00 [1.00; 5.00]	1.00 [1.00; 5.00]	2.00 [1.00; 10.0]	<0.001
Kidney disease:				0.565
No	974 (99.2%)	852 (99.3%)	122 (99.0%)	
Yes	8 (0.77%)	6 (0.7%)	2 (0.99%)	
Digestive tract bypass reconstruction:				0.154
No	727 (74.1%)	640 (74.60%)	84 (67.70%)	
Yes	255 (25.9%)	218 (25.40%)	40 (32.30%)	
Operation duration	210 [170; 274]	205 [165; 270]	260 [195; 295]	<0.001
TC (mmol/L)	4.28 [3.55; 5.05]	4.32 [3.63; 5.06]	3.83 [2.92; 4.93]	0.001
TG (mmol/L)	1.23 [0.92; 1.70]	1.24 [0.93; 1.70]	1.12 [0.86; 1.74]	0.157
HbA1C (%)	6.10 [5.50; 6.80]	6.00 [5.50; 6.70]	6.40 [5.50; 7.20]	0.044
BMI (kg/m^2^)	22.5 [20.4; 24.8]	22.5 [20.5; 24.9]	22.0 [19.6; 24.7]	0.089
Preoperative hypoglycemic administration methods:				<0.001
Others	888 (90.4%)	802 (93.50%)	86 (69.40%)	
Subcutaneous	87 (8.87%)	51 (5.94%)	36 (29.03%)	
Taken orally	7 (0.71%)	5 (0.58%)	2 (1.60%)	
Intraoperative hypoglycemic administration methods:				0.003
No	976 (99.4%)	856 (99.77%)	120 (96.77%)	
Bolus insulin	6 (0.60%)	2 (0.23%)	4 (3.23%)	
Hypoglycemic agents were used 1 day after surgery:				<0.001
Intravenous infusion	933 (95.0%)	826 (96.30%)	107 (86.30%)	
Subcutaneous injection	49 (4.99%)	32 (3.70%)	17 (13.70%)	
Preoperative nutritional support mode:				0.143
No	126 (12.83%)	112 (13.05%)	14 (11.30%)	
Parenteral	254 (25.86%)	210 (24.50%)	44 (33.70%)	
Enteral	66 (6.72%)	58 (6.76%)	8 (6.45%)	
Enteral+Parenteral	536 (54.6%)	478 (55.7%)	58 (46.77%)	
Preoperative fasting time:				<0.001
>24 h	834 (84.9%)	777 (90.60%)	57 (45.97%)	
≤24 h	148 (15.1%)	81 (9.40%)	67 (54.03%)	
The standard deviation of blood glucose levels over multiple days	2.04 [1.40; 2.86]	1.97 [1.37; 2.73]	2.33 [1.86; 3.20]	<0.001
Maximum range of blood glucose fluctuations over multiple days	6.60 [4.00; 9.88]	6.30 [3.90; 9.50]	8.50 [6.00; 13.1]	<0.001
Blood glucose fluctuation on the day of surgery	−1.00 [−1.00; 0.5]	−1.00 [−1.00; 0.21]	0.85 [−1.00; 2.8]	<0.001
The maximum amplitude of blood glucose fluctuation on the day of surgery	0.00 [0.00; 1.00]	0.00 [0.00; 0.30]	1.20 [0.00; 4.70]	<0.001

TC, total cholesterol; HbA1c, glycated hemoglobin; TG, triglyceride; BMI, body mass index.

**Figure 1:**
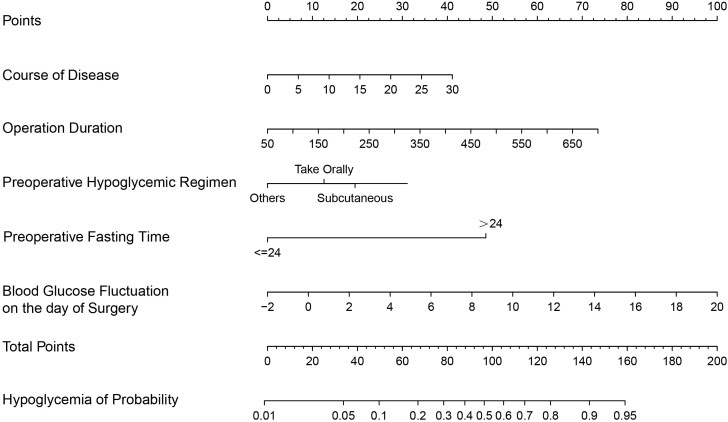
A predictive nomogram for hypoglycemia following gastrointestinal tumor surgery in patients with T2DM.

Following multivariate logistic regression analysis, five predictors—duration of diabetes, operation duration, preoperative fasting time, preoperative hypoglycemic administration methods (subcutaneous), and blood glucose fluctuation on the day of surgery—were identified and included in the predictive model of hypoglycemia risk following gastrointestinal tumor surgery (**Table 3**). A nomogram of hypoglycemia following gastrointestinal tumor surgery in patients with T2DM was developed based on logistic regression analysis results and is presented in **Figure 1**.

**Table 3:** Multivariate analysis of influencing factors on the risk of hypoglycemia after gastrointestinal tumor surgery in patients with T2DM.

Variables in the equation model	B	SE	Wald	Odds ratio (OR)	95% CI*	*P* value
Duration of diabetes	0.064	0.022	8.752	1.067	1.022–1.113	0.003
Operation duration	0.005	0.001	16.137	1.005	1.003–1.008	0.000
Preoperative hypoglycemic administration methods			7.291			0.026
Subcutaneous	0.915	0.342	7.148	2.497	1.277–4.883	0.008
Taken orally	0.591	0.951	0.386	1.806	0.280–11.652	0.534
Preoperative fasting time	2.282	0.266	73.640	9.795	5.817–16.496	0.000
Blood glucose fluctuation on the day of surgery	0.214	0.060	12.502	1.238	1.100–1.394	0.000
Constant	−4.369	0.415	110.963	0.013		0.000

*CI, confidence interval.

**Figure 2:**
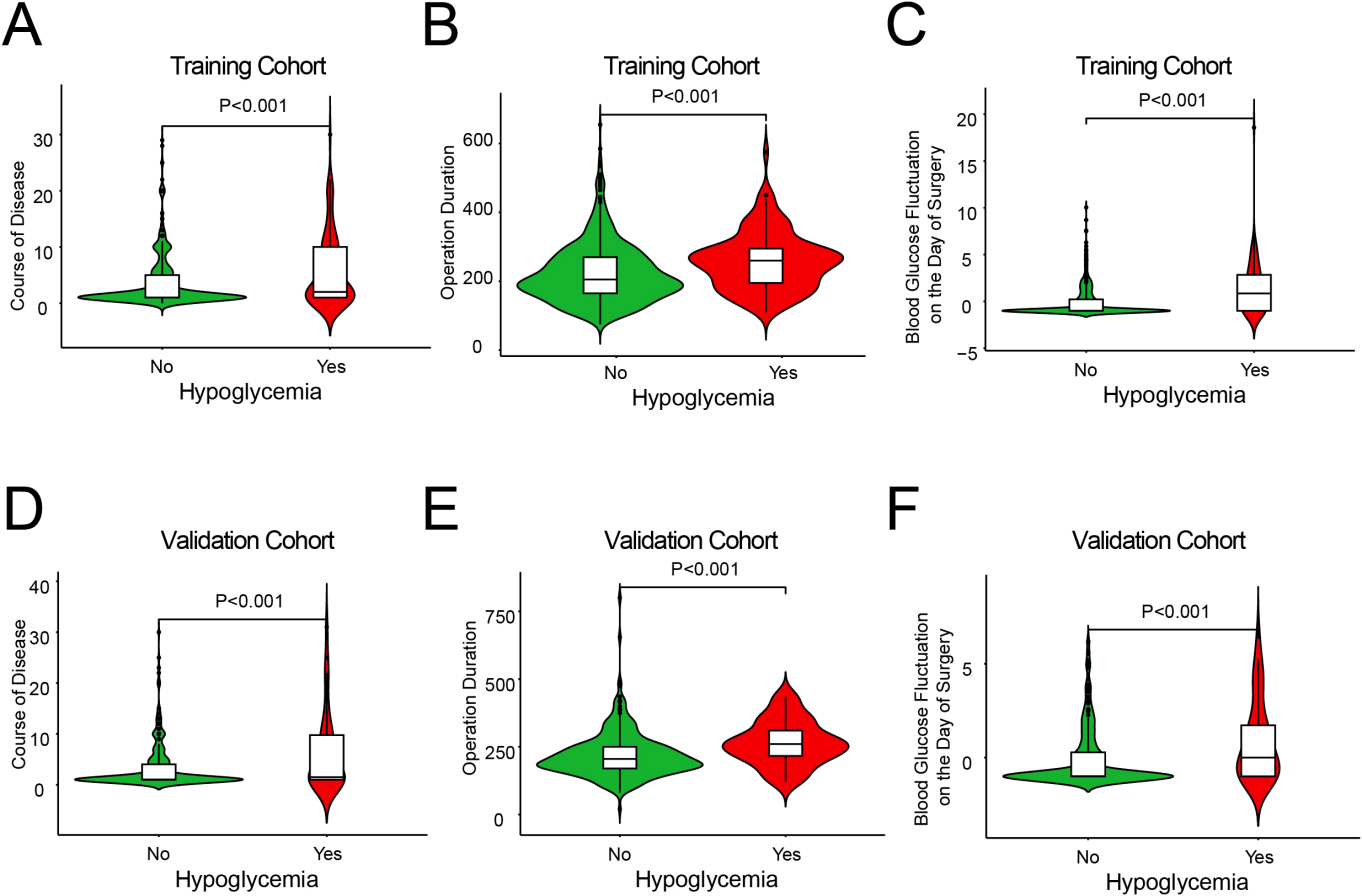
The distribution of continuous predictor variables between the groups with and without hypoglycemia. Plots **(A, D)** show course of disease, **(B, E)** show operation duration, and **(C, F)** show blood glucose fluctuation on surgery day.

In addition, we used violin plots to illustrate the distribution of data for three different influencing factors of hypoglycemia in patients with T2DM who underwent gastrointestinal tumor surgery, thereby verifying the robustness of the model on an independent dataset. We found that the distribution of these variables was significantly different between patients who experienced hypoglycemia and those who did not (*P* < 0.001) (**Figure 2**). In summary, these violin plots demonstrate that in both the training and validation cohorts, patients with a longer duration of diabetes, longer surgical duration, and more significant blood glucose variability on the day of surgery were more likely to experience hypoglycemia following gastrointestinal tumor surgery. These factors may be potential risk factors for postoperative hypoglycemia.

### 3.3 The performance of development and validation cohorts

**Figure 3:**
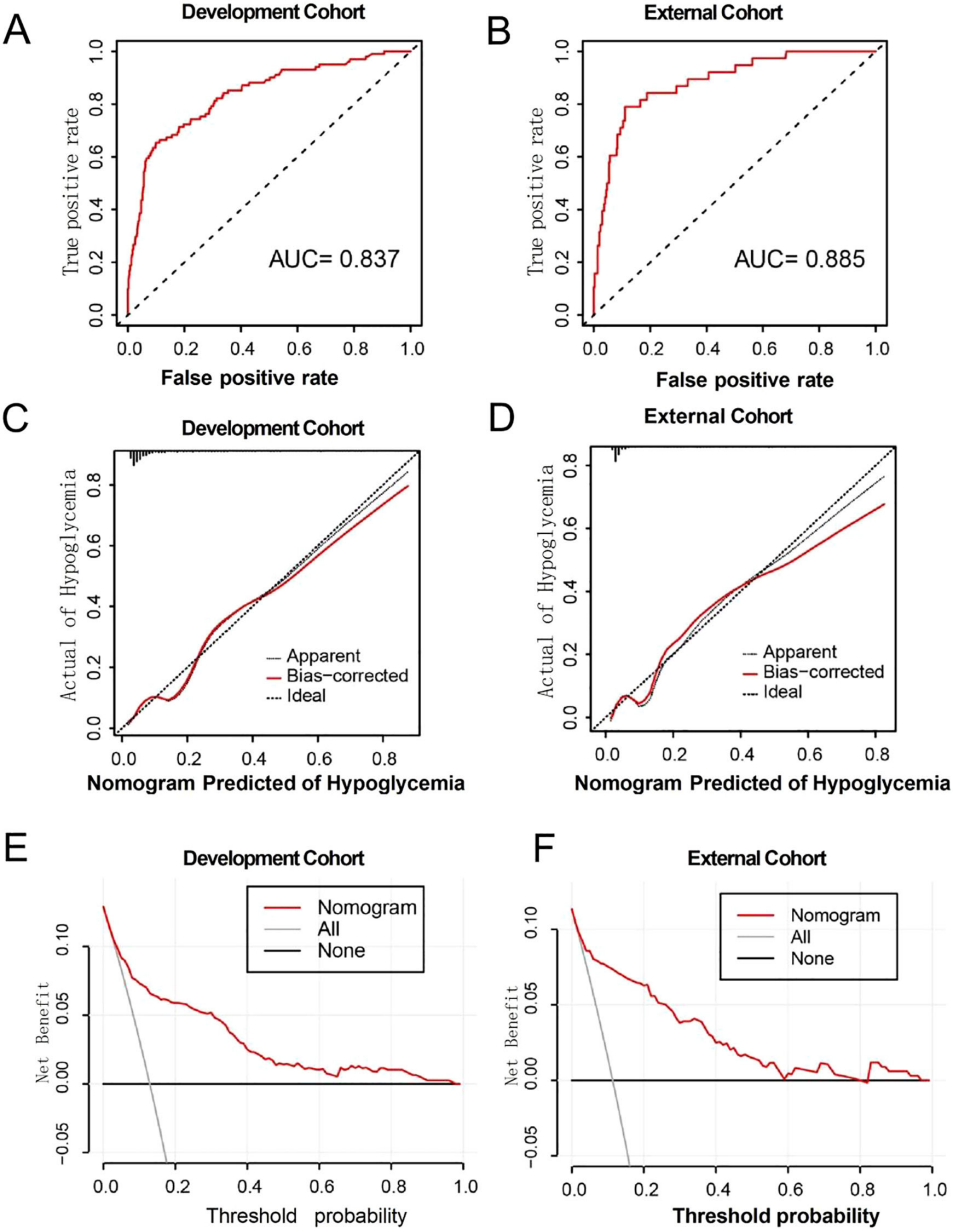
**(A, B)** ROC curves for development and external cohort. **(C, D)** Calibration curve for development and external cohort. **(E, F)** The decision curves for development and external cohort.

The area under the ROC curve (AUC) for predicting hypoglycemia was 0.837 (95% CI, 0.792–0.882) and 0.885 (95% CI, 0.829–0.941) in the development and validation cohorts, respectively (**Figures 3A, B**), indicating that the model demonstrated good discrimination ability in the validation cohort. The calibration curves revealed a favorable alignment between the predicted and observed cohort probabilities (**Figures 3C, D**). According to the decision curve analysis, when the model’s threshold is set between 6% and 85%, the decision curve lies above the “none” and “all” lines (**Figures 3E, F**). This indicates that, within this range, the model provides a positive net benefit.

## 4 Discussion

### 4.1 Model overview and clinical significance

Our study showed an incidence of 12.6% for hypoglycemia after gastrointestinal tumor surgery in patients with T2DM, which was similar to the report of Huiwu et al. (24). In the data we collected, it was found that about 30% of hypoglycemia occurred on the 2nd to 8th day after surgery. Therefore, it is important to evaluate high-risk patients using this nomogram on the 1st day after surgery and strengthen blood glucose management to reduce its incidence.

This study conducted an in-depth analysis of multiple risk factors associated with hypoglycemia after gastrointestinal tumor surgery in patients with T2DM. Ultimately, we found that the five risk factors were duration of diabetes, duration of the operation, preoperative fasting time, preoperative hypoglycemic administration methods, and blood glucose fluctuation on the day of surgery. Therefore, a predictive model for postoperative hypoglycemia risk in patients with T2DM was constructed; its predictive performance was evaluated using a receiver operating characteristic (ROC) curve, calibration curve, and decision curve analysis. The results show that the column chart has the characteristics of prediction accuracy, consistency, and clinical applicability.

### 4.2 Key predictive factors and clinical implications

#### 4.2.1 Duration of diabetes

Our study found that a longer duration of diabetes is an independent risk factor for hypoxemia after gastrointestinal tumor surgery in patients with T2DM. Compared with patients with diabetes lasting <10 years, patients with diabetes lasting ≥10 years had a 2.736-fold increased risk of hypoglycemia. This is consistent with previous research (27, 28). It was suggested that special attention should be paid to patients with long-term diabetes after surgery, and effective preventive measures should be taken to reduce the risk of hypoglycemia. Some measures could include increasing the frequency of blood glucose monitoring, and the medication regimen should be adjusted promptly to reduce the risk of postoperative hypoglycemia. Collaboration of multidisciplinary teams was essential for developing and implementing blood glucose management strategies (29).

#### 4.2.2 Operation duration

Our study found that longer operation duration was an independent risk factor for postoperative hypoglycemia. Prolonged surgery leads to increased fasting, which may further deplete the patient’s glucose reserves, increasing the risk of hypoglycemia (30). Studies (31, 32) have shown that patients using Ringer’s solution, isotonic NaCl solution, and amino acid supplement during operation are more prone to hypoglycemia, and intraoperative glucose supplement is an effective measure to prevent hypoglycemia. Therefore, blood glucose monitoring is crucial during surgery, especially for patients undergoing prolonged surgical procedures. Continuous blood glucose monitoring (CGM) systems are recommended to achieve continuous tracking and timely response to patients’ blood glucose levels. In addition, patients may delay eating and drinking due to discomfort such as pain, nausea, and vomiting after surgery, all of which may further affect their blood glucose levels. Therefore, it was imperative to reduce the risk of hypoglycemia through close monitoring of blood glucose during and after surgery and ensure the safety and comfort of patients.

#### 4.2.3 Duration of fasting

Our study demonstrated that the duration of fasting before surgery was a significant factor in the development of hypoglycemia. Preoperative fasting was a standard preparation measure before gastrointestinal tumor surgery, mainly to reduce the risk of intraoperative and postoperative complications (30). However, our study suggested an association between prolonged fasting before surgery and an increased risk of hypoglycemia: prolonged fasting might interfere with patients’ metabolic status and insulin sensitivity. These changes might reduce the level of insulin in the body, which increases the risk of hypoglycemia (32). All of the above factors might cause blood glucose fluctuations in patients before and on the day of surgery, which in turn leads to the risk of hypoglycemia after surgery. In our study, we included 1280 patients with gastrointestinal tumors, of whom 1089 (about 85.1%) had fasted for more than 24 hours before surgery. This phenomenon was primarily attributed to the patient’s gastrointestinal dysfunction or the anticipation of the surgery’s complexity. With the promotion of rapid rehabilitation surgery (30), the preoperative fasting time has been significantly shortened. Several prospective, randomized controlled studies (33, 34) have confirmed that consuming oral carbohydrate liquids before surgery can shorten the length of hospital stay and reduce the incidence of postoperative complications. However, further research is needed to determine whether it is suitable for patients with diabetes. Additionally, uncertainty about the timing of surgery may cause patients to extend their fasting time to accommodate potential changes in the surgery schedule. Therefore, we needed to reasonably plan the preoperative fasting time and formulate a nutritional treatment plan that could effectively reduce the risk of hypoglycemia.

#### 4.2.4 Preoperative hypoglycemic administration methods (subcutaneous)

Patients undergoing surgery for gastrointestinal tumors are typically provided with nutritional support during the perioperative period (35). Among the 1280 cases in our study, preoperative nutritional support accounted for 87.6%, including 343 cases (26.8%) of preoperative parenteral nutrition and 690 cases (53.9%) of preoperative parenteral and enteral combined nutrition.

Hyperglycemia is extremely common in both diabetic and non-diabetic patients receiving nutritional therapy. Compared with enteral nutrition (EN), patients treated with parenteral nutrition (PN) are more likely to have significant hyperglycemia because PN bypasses glucagon-like peptide-1 and gastric inhibitory polypeptide in the gastrointestinal tract, resulting in the loss of the incretin effect (36). Numerous studies (37) have confirmed an association between enteral nutrition and parenteral nutrition, which is associated with hyperglycemia and poor clinical outcomes. In addition, patients with severe or postoperative surgery often suffer from hyperglycemia due to stress, so strict monitoring of blood glucose and intensive insulin therapy strategies would be implemented. However, intensive blood glucose control may also be associated with an increased risk of hypoglycemia (38). In the data analysis of this study, we found that 8.7% of patients used subcutaneous insulin injections before surgery. In the multifactor regression model, this administration mode was identified as an independent factor affecting the risk of postoperative hypoglycemia, which was significantly correlated with the occurrence of hypoglycemic events. We recommend using an insulin pump in combination with a continuous blood glucose monitoring system to monitor blood glucose fluctuations and adjust the treatment plan promptly, ensuring blood glucose control while reducing the occurrence of hypoglycemia.

#### 4.2.5 Fluctuation of blood glucose on the day of surgery

Blood glucose fluctuations encompass short-term diurnal and intraday variations, as well as long-term variations in glycosylated hemoglobin. There is a close relationship between the risk of hypoglycemia and blood glucose fluctuations. We use standard deviation of blood glucose (SDBG) to supplement changes in blood glucose fluctuations. The standard deviation of blood glucose (SDBG) is a key measure of blood glucose dispersion. A higher SDBG reflects greater glycemic variability, while a lower SDBG indicates more stable blood glucose levels. Our study found that higher SDBG was associated with an increased risk of postoperative hypoglycemia, especially blood glucose fluctuations on the day of surgery. Compared with diabetics with an SDBG <3.0 mmol/L, diabetics with an SDBG ≥3.0 mmol/L had a 2.897-fold increased risk of postoperative hypoglycemia. This is similar to the study by Yuan SJ et al. (38). The fluctuation of blood glucose on the day of surgery may be a key influencing factor of postoperative hypoglycemia. This factor had not been mentioned in previous studies. Nervous psychological state before surgery, fasting before surgery, and surgery may bring significant blood glucose fluctuations, which can easily cause hypoglycemia. Some measures, including the reasonable adjustment of hypoglycemic treatment before surgery and the strengthening blood glucose monitoring before surgery, can reduce the occurrence of hypoglycemia. Nurses, in particular, need to be aware of the potential discomfort that preoperative patients may experience due to the surgery. Nurses can help alleviate patients’ anxiety by explaining the surgical process and providing comfort and emotional support. Additionally, our study observed that anesthesiologists regularly monitored arterial blood gases to obtain glucose values and administered intravenous insulin injections promptly to control blood glucose levels effectively. The occurrence of hypoglycemia was closely related to the speed and amplitude of blood glucose decline. In the results of the univariate analysis, we also found that whether insulin was used during the operation was a significant factor in the occurrence of hypoglycemia. Nurses should pay special attention to the intraoperative blood glucose status and implement more frequent blood glucose monitoring to be prepared to respond promptly to any signs of hypoglycemia.

### 4.3 Implications for clinical practice

Emaciation in patients with gastrointestinal tumors results from multiple factors, which may be related to the energy consumption of the tumor itself, the reduction of nutritional intake of patients, metabolic disorders, psychological factors, and other aspects. Although body mass index was not a factor in hypoglycemia after gastrointestinal tumor surgery in T2DM patients in our study, multiple studies have reported a negative correlation between body mass index and hypoglycemia (39, 40). This may be the reason why patients with gastrointestinal tumors are more prone to hypoglycemia than other populations. Therefore, clinical attention should still be paid to postoperative hypoglycemia in T2DM patients with low body mass index. In addition, several studies have confirmed that gastric bypass surgery can significantly improve blood glucose control in patients with T2DM, achieving therapeutic effects (19, 41, 42). In this study, we did not find a significant correlation between different methods of digestive tract reconstruction surgery and the risk of postoperative hypoglycemia. This outcome might stem from the restricted follow-up period in our research. Some studies indicate that patients with T2DM may experience hypoglycemic events in the short term (within a few days to a few weeks) after gastric tumor surgery and that the risk of hypoglycemia may be further increased in the more extended postoperative period (1 to 12 months) due to dietary and lifestyle adjustments (43, 44). Thus, in future research, we plan to extend the follow-up period and systematically monitor the long-term incidence of hypoglycemia. This approach will allow for a comprehensive assessment of whether different reconstructive surgical methods affect the risk of hypoglycemia over time, particularly during the critical postoperative adaptation phase. In addition, healthcare professionals are encouraged to monitor the blood glucose levels in T2DM patients discharged after gastrointestinal tumor surgery. Enhanced patient education on home-based self-care can help patients better manage their condition and reduce the risk of hypoglycemia.

Continuous glucose monitoring (CGM) is highly valuable in perioperative glycemic management. Unlike traditional blood glucose testing, CGM provides continuous real-time data with measurements every 5 minutes, uncovering missed glucose fluctuations. This allows timely interventions to prevent complications. CGM also quantifies glycemic variability using the coefficient of variation (CV), helping to identify high-risk patients. Personalized strategies guided by CV can reduce adverse effects of glucose swings. Moreover, CGM improves clinical outcomes by detecting abnormalities during critical periods like preoperative fasting and the early postoperative phase, enabling targeted insulin and dietary adjustments (31, 38, 45). In summary, CGM enhances hypoglycemia management precision through high-frequency tracking, variability analysis, and real-time alerts. It provides crucial data for early interventions, minimizing hypoglycemia-related morbidity and improving patient outcomes. This technology has the potential to be widely adopted in clinical practice.

### 4.4 Study limitations

Despite the strengths of our study, several limitations should be acknowledged. First, the retrospective design limits the ability to establish causality. While our model identifies associations between predictors and hypoglycemia occurrence, prospective studies are necessary to confirm these findings and refine the model. Second, the lack of data from other institutions restricts accurate external validation. Although internal validation was performed, external validation in diverse populations and varied care settings is needed to confirm the model’s universal applicability across different clinical environments. To enhance the external validity and broaden the applicability of our model, we will conduct a multicenter study incorporating diverse populations from various geographical regions, ethnic backgrounds, and medical environments. Our research design will include a wide array of patient characteristics and variables to better reflect population diversity and strengthen the model’s generalization capability.

Additionally, this study did not consider factors related to tumor origin. Future research should expand the sample size to investigate this aspect. This includes using subgroup analyses to examine how primary tumor location, TNM staging, metastasis, and combination therapies (such as chemotherapy with or without radiotherapy or immunotherapy) affect blood glucose homeostasis.

## 5 Conclusions

We presented a nomogram to predict the risk for hypoglycemia after gastrointestinal tumor surgery in patients with T2DM. This tool proved efficient and precise for initial screening. This new nomogram could help surgical nurses assess individual patient risks and implement appropriate preventive measures.

## Data Availability

The raw data supporting the conclusions of this article will be made available by the authors, without undue reservation.
